# Organophosphate poisoning of Hyacinth Macaws in the Southern Pantanal, Brazil

**DOI:** 10.1038/s41598-021-84228-3

**Published:** 2021-03-10

**Authors:** Eliane C. Vicente, Neiva M. R. Guedes

**Affiliations:** FUNDECT/CAPES, Projeto Morcegos Brasileiros, Instituto Arara Azul, MDR Uniderp University, Campo Grande, Brazil

**Keywords:** Zoology, Environmental sciences

## Abstract

The populations of hyacinth macaws (*Anodorhynchus hyacinthinus*), an emblematic species, have suffered declines due to many environmental factors. The Hyacinth Macaw Institute’s actions are showing positive outcomes for the conservation of *A. hyacinthinus*. However, environmental issues, such as fires and deforestation due to inefficient and unsustainable cattle ranching practices, are a threat to the biodiversity. Another major threat is the reckless use of pesticides. The objective of this manuscript is to describe the findings, in the Pantanal, of three dead hyacinth macaws and to investigate their cause of death and conservation implications. A necropsy was conducted on two individuals and biological samples were collected and sent to conduct toxicological exams to test for organophosphates, organochlorines, and carbomates. Compatible with other findings, results showed a highly dangerous level of organophosphate, 158.44 ppb. We describe for the first time, a rare, isolated but unusual mortality event associated with organophosphate pesticide poisoning of hyacinth macaws. Mortality reports for bees and other bird species on how the improper use of pesticides can potentially cause the contamination of food and water resources are discussed. These factors are antagonistic to long-term efforts to preserve wildlife and carry out other conservation efforts in Brazil’s southern Pantanal.

## Introduction

The Hyacinth Macaw Institute (HMI = Instituto Arara Azul) is part of a 30 year old conservation program in which, at the start of the project in 1987, it was estimated there were approximately 2500 wild hyacinth macaws^1^. Currently, as result of a successful conservation program in the southern Brazilian Pantanal of Mato Grosso do Sul, the wild population is estimated at approximately 6.500 individuals^2,3^.

The Pantanal, located in central South America, is one of the world’s largest continuous freshwater floodplains in the world. It supports a highly productive and diverse assemblage of neotropical flora and fauna, provides important regional and global ecosystem services, and as a result, it was designated by UNESCO as a Biosphere Reserve in 2002^4^. A large portion of this ecosystem consists of private properties, whose main economic activities include extensive livestock production, tourism and fishing. Nevertheless, the Pantanal has one of the highest conservation practices in Brazil, where numerous threatened animal species can still be seen, such as jaguars, tapirs, giant anteaters and hyacinth macaws.

The hyacinth macaw is specialized in its choice of food and nest site, and therefore it has experienced population declines due to habitat degradation, such as deforestation and forest fragmentation, and illegal wildlife trade^5–7^.

The HMI has successfully reduced the number of macaw seizures for the illegal wildlife trade primarily due to environmental awareness programs that focuses on educating the local population, and the expansion of effective patrolling^1,3,5,6^.

The long-term monitoring and continued research studies indicate numerous other factors that are also important threats to the hyacinth macaws. Besides deforestation, infrastructure (e.g. power lines) and pollutants (visual, audible and chemical)^8^ increase the mortality rate of juvenile birds.

Crop protection products have been adapted to minimize impact on human and animal health. However, products are more diverse, with less residual impact, but they still cause significant damage, which can sometimes go undetected^9^.

The use of pesticides can affect wildlife directly and indirectly. Some animals can be directly affected during pesticide application, because they consume the contaminated food and water, or by secondary poisoning from the residual pesticide effects on air, water and soil contamination. The residual effects of pesticides can promote behavioral changes in many organisms, and affect important developmental stages or aspects of their biological cycle, especially reproduction^10–12^.

The increase in the release of pesticides in Brazil since 2019 is a serious concern, especially with the current knowledge of pesticide residual effects on the environment. Pesticide residue impact have affected several animal groups, especially bees, one of the most studied groups regarding the consequences and damages caused by synthetic or phytomolecular poisons^13–15^.

The residual effects of organophosphates are small when compared to other pesticides such as organochlorines and carbamates. However organophosphates can reside in the environment for at least three years, and their high aqueous solubility allows them to be washed away quickly, increasing their contamination potential ^16,17^.

Our objective for this study was to describe the findings of the three dead hyacinth macaws encountered in the Pantanal, and investigate the cause of mortality and its conservation implications.

## Materials and methods/procedures

**Figure 1:**
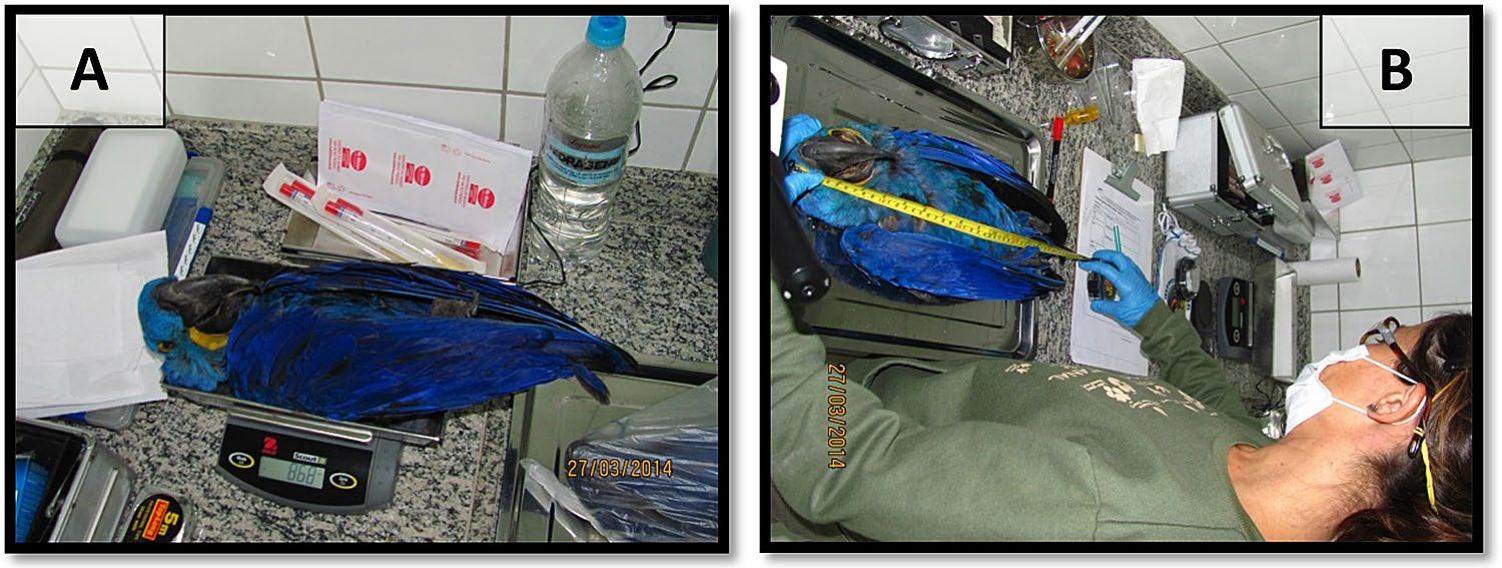
In (**A**) the individual marked 2014-02 being weighted and in (**B**) individual 2014-03. Photo: Carlos Cézar Corrêa.

In 2014 three hyacinth macaws were found by a landowner who is also a veterinarian, from a remote farm in the Pantanal of Rio Negro, Mato Grosso do Sul, Brazil. One of the individuals had recently died and was not sampled. Once the specimens were sent to HMI, necropsied individuals received identification numbers (ID 2014–02 and 2014–03). The state of the specimens in terms of organic and physiological condition was similar, but one was in a more advanced state of decomposition (Fig. 1A,B).

Visceral organs such as the liver, spleen and stomach (pro-ventricle and mechanical stomach—gizzard), were sent to a reference Toxicological Service Center for analysis—CEATOX of Biosciences at the Sao Paulo State University “*Julio de Mesquita Filho*” (UNESP—Botucatu, SP).

Liver samples were analyzed for the identification and quantitative determination of organophosphate (analysis no. 180/2014) and organochlorines (analysis no. 181/2014) using gas chromatography and a capillary column, coupled to an electron capture detector. The contents of the gizzard were analyzed to determine and identify the presence of carbamates (analysis no. 182/2014) with liquid chromatography (HPLC) and an ultraviolet detector^17^.

The toxicological data obtained by the experts' findings allowed reliable conclusions about the cause of death of necropsied animals. The information obtained is descriptive (case study).

## Results and discussion

Of the three investigated hyacinth macaws, one of the individuals had recently died and was not sampled. This particular specimen showed bodily fluid extravasation into its oral cavity and nose. The two other individuals were found alive but neurological symptoms were observed, and soon they died in the same manner, and sent to HMI. These occurrences are unusual, isolated and rare for hyacinth macaws. Results of the laboratory exams are shown in Table 1, where it is possible to observe a high concentration of Mevinsphos (Phosdrin) with 158.5 ppb level. The data obtained identified chemical factors that promote neural alteration^18^. Externally we observed some bruises in different parts of the body of the two individuals which may have been due to ataxia caused by the toxic agent.

**Table 1:** Results of toxicological analysis of the liver and gizzard of two hyacinth macaw specimens performed by CEATOX – UNESP, Botucatu, SP.

Samples and type of pesticisdes tested		
180/2014 Organophosphates	181/2014 Organochlorines	182/2014 Carbamates
Sample: liver Mevinfos (Phosdrin): 158,4 ppb	Sample: liver < 1,0 ppb	Sample: gizzard contents N.D. = < 1 ppb

**Figure 2:**
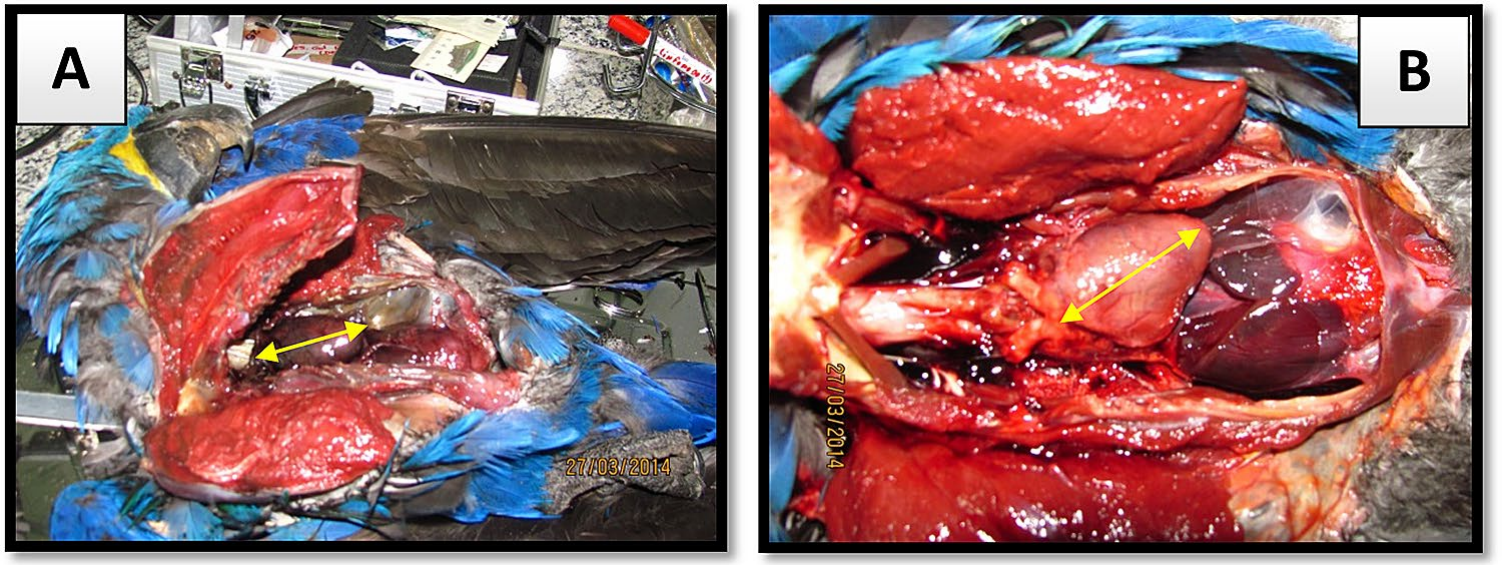
Differences in visceral coloration of two individuals. Heart length: In [(**A**) 2014-02 = 41.3 mm and in (**B**) 2014-03 = 41.6 mm]. Photo: Eliane Vicente.

Internally there were very significant differences in their state of decomposition. Specimen 2014–02 showed 24 h more decay than the predicted time of death, and yet both had intact airbags and visceral organs. The color and texture of organs were used to determine the time difference between the two decomposing animals. Besides the differences of decomposition time, both lungs showed signs of hemorrhagic edema, leading to the likely cause of death being cardiac arrest (Fig. 2A,B).

**Figure 3:**
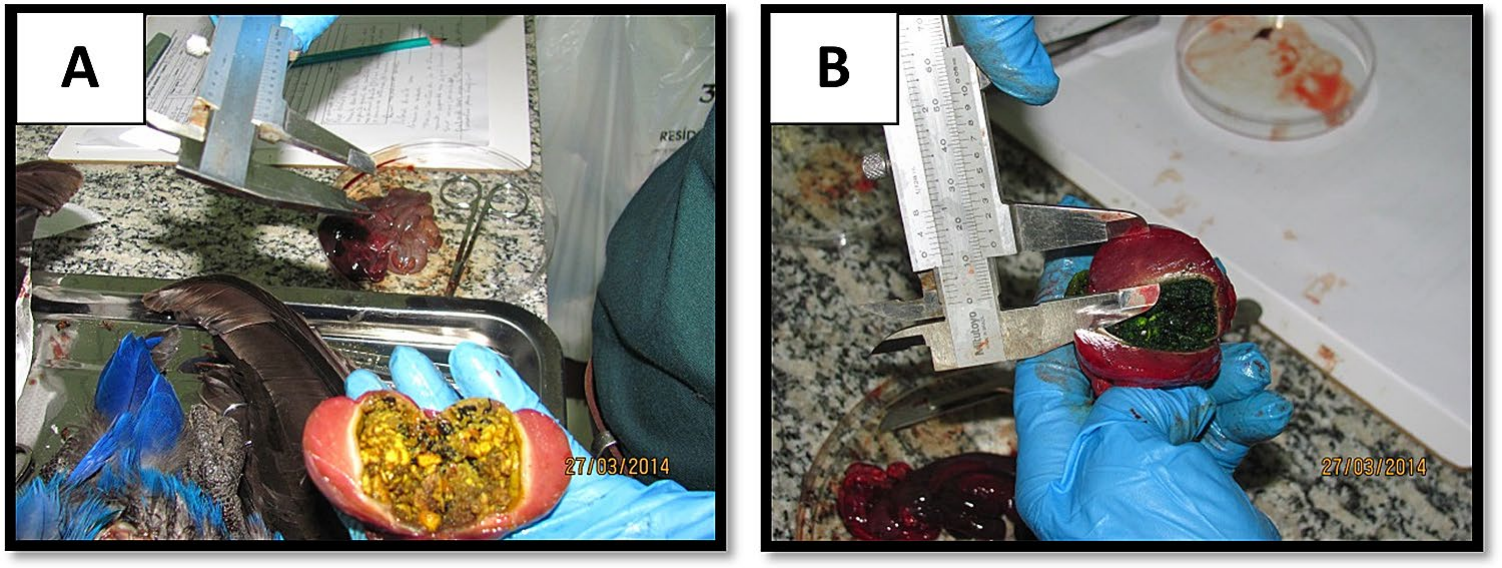
The gizzard specimens [(**A**) 2014-02 and (**B**) 2014-03]. Photo: Carlos Cézar Corrêa.

The estimated time of death was established by evaluating tissue hardness. The animal 2014–02 indicated a typical gizzard but, inside the gizzard of specimen ID 2014–03 there was grass with visible chlorophyll indicating an attempt to self-medicate (Fig. 3A,B).

Results of the samples analyzed confirm the poisoning by chemical factors that promote neural alteration. Although the gizzard content was atypical in individual 2014–03, its toxicological analysis was negative for carbamate and organochlorine (in 181.182/2014- CEATOX). The analysis indicated organophosphate liver poisoning (180/2014- CEATOX (Table 1). This toxicological result is consistent with the symptoms reported by the veterinarian who collected the animal and the data obtained from the necropsy report. With regard to Phosdrin's LD50, according to the World Health Organization (WHO), 1 ppb is the lethal dose for laboratory animals, such as rats, and depending on the different paths of exposure and absorption, whether oral or dermal, above 50 ppb is considered highly dangerous^19^. At the moment, there are no scientific studies or reports for domestic or wild animals. However, the detection of Phosdrin 158.4 ppb in the liver of the macaws is compatible to an intoxication diagnosis, and confirms acute death, due to poisoning of these macaws.

However, we believe that this event is uncommon, because in 30 years of research in the Pantanal we have never found a similar case with this species or other large macaws. We do not have evidence of regular use of this pesticide in the Pantanal, so most likely this application was punctual, and improperly or even carelessly applied. Furthermore, it cannot be confirmed that the macaws were intoxicated on this farm or on another property, because contaminated macaws were only reported from this property.

According to the literature, organophosphate contamination includes neurological symptoms that compromise the respiratory system and the cardiovascular center. Like any neurotoxic agent, it impairs motor function that leads to uncoordinated movements, clearly observed by ataxia^17,20,21^. As mentioned earlier, it must be considered that the various organophosphates are highly soluble and easily absorbed by mucous membranes. There is strong evidence that contaminated crops and water resources leads to an increased risk of mortality associated with agro toxins and pesticide poisoning^17^.

The 1989 Pesticide Act regulated the use, production and inspection of chemicals and pesticides to minimize the risks and potential environmental contamination and poisoning of farmers and/or people, directly or indirectly exposed to these products^22^. In July 2002, a Federal Law was reformulated (no. 9974, improved from the 1989 pesticide law).

Evidence of organophosphate contamination was identified in the intestinal tracts of the Swainson’s hawks. In 1996, mortality reports of more than eight thousand Swainson’s hawk (*Buteo swainsoni*) specimens were found in the Argentinian Pampas^23^. Contamination of organochorines were reported between 2015 and 2017 in southern Brazil where researchers detected the presence of organochlorines in feathers of three raptor species (*Phalcoboenus chimango*, *Milvago chimachima* and *Caracara plancus*) ^24^. The samples were obtained from live animals and the concentrations of organochlorine were higher than reported from other species of raptors. *Caracara plancus* samples showed the highest concentrations of organochlorine probably because this species feeds mostly in urban and agricultural environments^24^.

In India, (2000), fifteen *Grus antigon* and three *Grus grus* were found dead in a field next to an area where wheat seeds were sown the day before. Residues of the monocrotophos organophosphate insecticide were identified in the chemical analysis of seed samples, and in the bird's digestive tract that died from poisoning^25^.

A compilation of a 36 year analysis (1976 – 2012) of mortality factors for wild birds in the USA, list organophosphate compounds as the most common factor for adult bird mortality. These compounds have been found in more than 100 species of wild birds^26^.

The historical misuse of pesticides impacted Peregrine falcon reproduction and adult survival in North America and the British Isles, respectively^27^. The impact of pesticides on wild birds is also detected in other countries and continents, in which the diversity of birds decreases after the application of pesticides in a given area. Although most birds are susceptible to pesticide exposure, the symptoms and intensity of the effects will vary with the natural history of each species, and the behavior and physiology of each group^25^.

Even though the pesticides used today have less residual effects and are safer, we must still be cautious that the established safety levels are in accordance with the criteria established for human metabolism, and medium to large size livestock or pets. For captive animals, ingested food and water are controlled and generally safe from contamination. However, for wildlife, resources are not necessarily controlled or monitored.

In 2020 in only four Brazilian states, more than five hundred million bees were found dead by beekeepers. The main cause for this mortality was the use of neonicotinoid and fipronil pesticides, which are very lethal, and the continued spraying across different environments is leading to the extermination of many bee populations. Bees are important pollinators and essential for food production sectors all over the world. According to United Nations Food and Agriculture Organization (FAO), 75% of plants consumed by humans are dependent on bees^28^. Although birds have not been impacted as much as bees, they have adaptations that make them vulnerable and sensitive to pesticide contamination, and they have greater potential for exposure to all types of toxic agents. These adaptations include unique physiological patterns characterized by high metabolic rates, low weight and low body mass. These traits may be important for the future conservation of bird populations especially with the reduced restrictions of pesticide use in Brazil, including those banned for a long time in many countries in Europe, North America, and others.

Another feature of birds that makes them more susceptibility to poisoning is their thin integument and their preening behavior using their beak. This behavior causes chemicals on the feathers to come into contact with the integument and the digestive tract, which are high permeability tissues.

Free-ranging wildlife is constantly exposed to pesticides and can serve as indicators for human and environmental health. However, most of the effects on wild fauna are not observed, especially regarding mortality in nature, and therefore does not get reported^29^.

## Conclusion

It is not easy to find dead small animals, especially birds in their natural environment. In the Pantanal, depending on the type of vegetation and especially the vast ecological interactions with other species of fauna, it is even more difficult to find. In reality, it is rare when a person finds a carcass before a crab-eating fox or caracara. In this study case, once the owner found the three macaws, the HMI was immediately notified. For this reason it was possible to identify the cause of death, and subsequently suggest appropriate management strategies to prevent future deaths.

The toxicity of organophosphates and other pesticides indicate how potentially aggressive they are for the environment and wildlife. Therefore we recommend that the use of any organophosphate should not be applied in or adjacent environmental reserves and especially aquatic ecosystems such as the Pantanal.

## Supplementary Information

Supplementary Information[replace with the revised file]
