# Supplementary material for: Organophosphate poisoning of Hyacinth Macaws in the Southern Pantanal, Brazil
Source: Sci Rep. 2021 Mar 10;11:5602. doi: 10.1038/s41598-021-84228-3 (PMC7946944; doi:10.1038/s41598-021-84228-3)
Supplement: Supplementary file 1 — Supplementary Information[replace with the revised file] [file 41598_2021_84228_MOESM1_ESM.pdf]

Organophosphate poisoning of Hyacinth Macaws in the Southern Pantanal, Brazil

Eliane C. Vicente[1]; *Neiva M. R. Guedes[2]

*Correspondence to guedesneiva@gmail.com

RESULT OF TOXICOLOGICAL ANALYSIS 180/2014

ANALYSIS: Quantitative Determination of Organophosphates.

REQUEST DATA
Applicant: Instituto Arara Azul
CNPJ: 05.910.537/0001-02
Address: Rua Klaus Sthurk, 106 – Jardim Mansur – Campo Grande/MS/Brazil
Zip Code: 9051-660
Phone: +55 (67) 3222-1205

ANIMAL DATA:
Identification: Blue Macaws

ANALYZED SAMPLES: Liver

RECEIVED ON: 05/26/2014 **COMPLETED ON:** 07/02/2014

IDENTIFICATION TECHNIQUE: Gas chromatography, capillary column, coupled to an electron capture detector.

RESULT(S):

Organophosphate Insecticides	Liver
Coumaphos (Co-ral)	< 10,0 ppb
DDVP (Diclorvos)	< 10,0 ppb
Diazinon	< 10,0 ppb
Dimethoate (Cygon)	< 10,0 ppb
Ethion	< 10,0 ppb
Fenthion	< 10,0 ppb
Malathion	< 10,0 ppb
Methyl-parathion	< 10,0 ppb
Mevinfos (Phosdrin)	**158,4 ppb**

ppb = Parts per billion or µg/L

Note: The samples were collected and sent by the applicant.

Msc. Fábio Iachel da Silva
Chemist
CRQ 04136549

RESULT OF TOXICOLOGICAL ANALYSIS 181/2014

ANALYSIS: Quantitative Determination of Organochlorines

REQUEST DATA
Applicant: Instituto Arara Azul
CNPJ: 05.910.537/0001-02
Address: Rua Klaus Sthurk, 106 – Jardim Mansur – Campo Grande/MS/Brazil
Zip Code: 9051-660
Phone: +55 (67) 3222-1205

ANIMAL DATA:
Identification: Blue Macaws

ANALYZED SAMPLES: Liver

RECEIVED ON: 05/26/2014 **COMPLETED ON:** 07/02/2014

IDENTIFICATION TECHNIQUE: Gas chromatography, capillary column, coupled to an electron capture detector.

RESULT(S):

Organochlorines Insecticides	Liver
Aldrin	< 1,0 ppb
pp`-DDD	< 1,0 ppb
pp`-DDE	< 1,0 ppb
pp`-DDT	< 1,0 ppb
Dieldrin	< 1,0 ppb
Endosulfan-1	< 1,0 ppb
Endosulfan-2	< 1,0 ppb
Endrin	< 1,0 ppb
Heptachlor	< 1,0 ppb
Eptide heptachlor	< 1,0 ppb

ppb = Parts per billion or µg/L

Note: The samples were collected and sent by the applicant.

Msc. Fábio Iachel da Silva
Chemist
CRQ 04136549

RESULT OF TOXICOLOGICAL ANALYSIS 182/2014

ANALYSIS: Quantitative Determination of Carbamates

REQUEST DATA
Applicant: Instituto Arara Azul
CNPJ: 05.910.537/0001-02
Address: Rua Klaus Sthurk, 106 – Jardim Mansur – Campo Grande/MS/Brazil
Zip Code: 9051-660
Phone: +55 (67) 3222-1205

ANIMAL DATA:
Identification: Blue Macaws

ANALYZED SAMPLES: Gizzard content

RECEIVED ON: 05/26/2014 **COMPLETED ON:** 07/02/2014

IDENTIFICATION TECHNIQUE: Çiquid chromatography (HPLC) with ultraviolet detector

RESULT(S):

Carbamates	Gizzard content
Aldicarb	N.D.
Bendiocarb	N.D.
Carbaryl	N.D.
Carbofuran	N.D.
Propoxur	N.D.

N.D. < 0,001 µg/g

Note: The samples were collected and sent by the applicant.

Msc. Fábio Iachel da Silva
Chemist
CRQ 04136549
